# DWI-FLAIR Mismatch at MRI Versus Infarct-Penumbra Mismatch on CT Perfusion

**DOI:** 10.5334/jbsr.1906

**Published:** 2019-10-31

**Authors:** Margaux Collard, Thierry Duprez, Sanaa Jamali

**Affiliations:** 1 Cliniques Universitaires Saint Luc, BE

**Keywords:** acute ischemic stroke, MRI, CTA, CTP, mismatch

Among radiologists and clinicians, there may be confusion between the terms and the implications of ‘MR-mismatch’ and ‘CTP-mismatch’ for hyperacute stroke management.

## Case report

**Figure 1:**
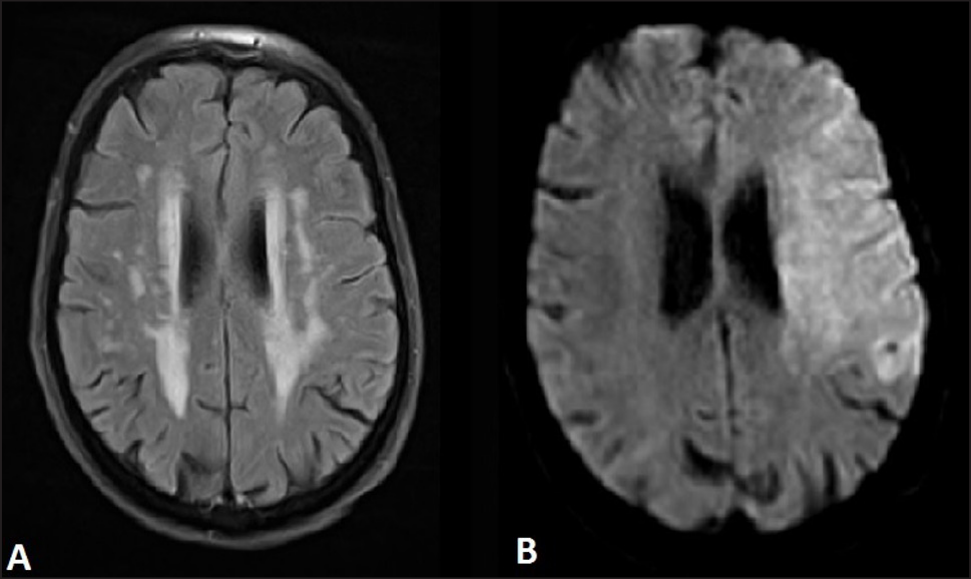
MRI of the brain with **(A)** axial FLAIR image showing leukoaraiosis and **(B)** axial DWI showing high signal intensity in the territory of left MCA.

A 90-year-old female patient was brought to our hospital by ambulance after her husband found her somnolent and mute upon waking. Upon arrival, neurological and physical examinations showed spastic flexion of the right arm and deviation of gaze to the left. A so-called ‘wake-up stroke procedure’ was triggered and brain magnetic resonance imaging (MRI), including fluid-attenuation inversion-recovery (FLAIR) and diffusion weighted imaging (DWI) acquisitions, was immediately performed (Figure 1). Axial FLAIR images showed only leukoaraiosis (Figure 1A). DWI demonstrated high signal intensity in the territory of left middle cerebral artery (MCA) at high b-value (Figure 1B) with decreased apparent diffusion coefficient values. MR-angiography was precluded by patient’s movements.

**Figure 2:**
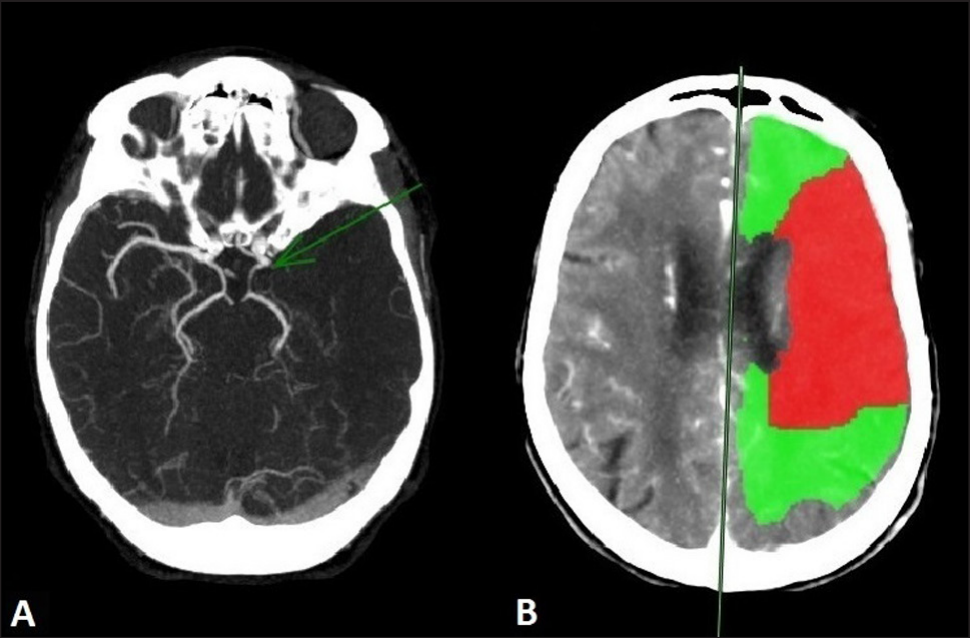
**(A)** CTA image showing proximal occlusion of the left MCA (green arrow) and **(B)** CTP image showing a mismatch between the infarcted core in red and the peripheral hypoperfusion in green.

Non-contrast CT (NCCT), CT-angiography (CTA), and CT-perfusion (CTP) were thereafter performed for angiographic assessment including carotid arteries, circle of Willis, and intracranial collateral circulation (Figure 2). NCCT showed loss of differentiation between grey matter and white matter in the left MCA territory and acute thrombotic hyperdensity within the MCA. CTA showed proximal occlusion of the left MCA (Figure 2A) and CTP showed a perfusion mismatch between infarcted core (Figure 2B, red area) and peripheral hypoperfusion (Figure 2B, green area) featuring the ischemic penumbra (Figure 2B).

IV thrombolysis was immediately initiated. Thrombectomy was not considered because of the patient’s age and other comorbidities.

## Comment

There is a common confusion about the terms ‘mismatch’ in hyperacute stroke management. The ‘MR-mismatch’ between positive DWI and negative FLAIR is a temporal indicator informing on the onset of ischemia of less than 4–5 hours. In turn, the ‘CTP-mismatch’ has anatomic value by imaging the ischemic penumbra as target for therapeutic interventions.

**Figure 3:**
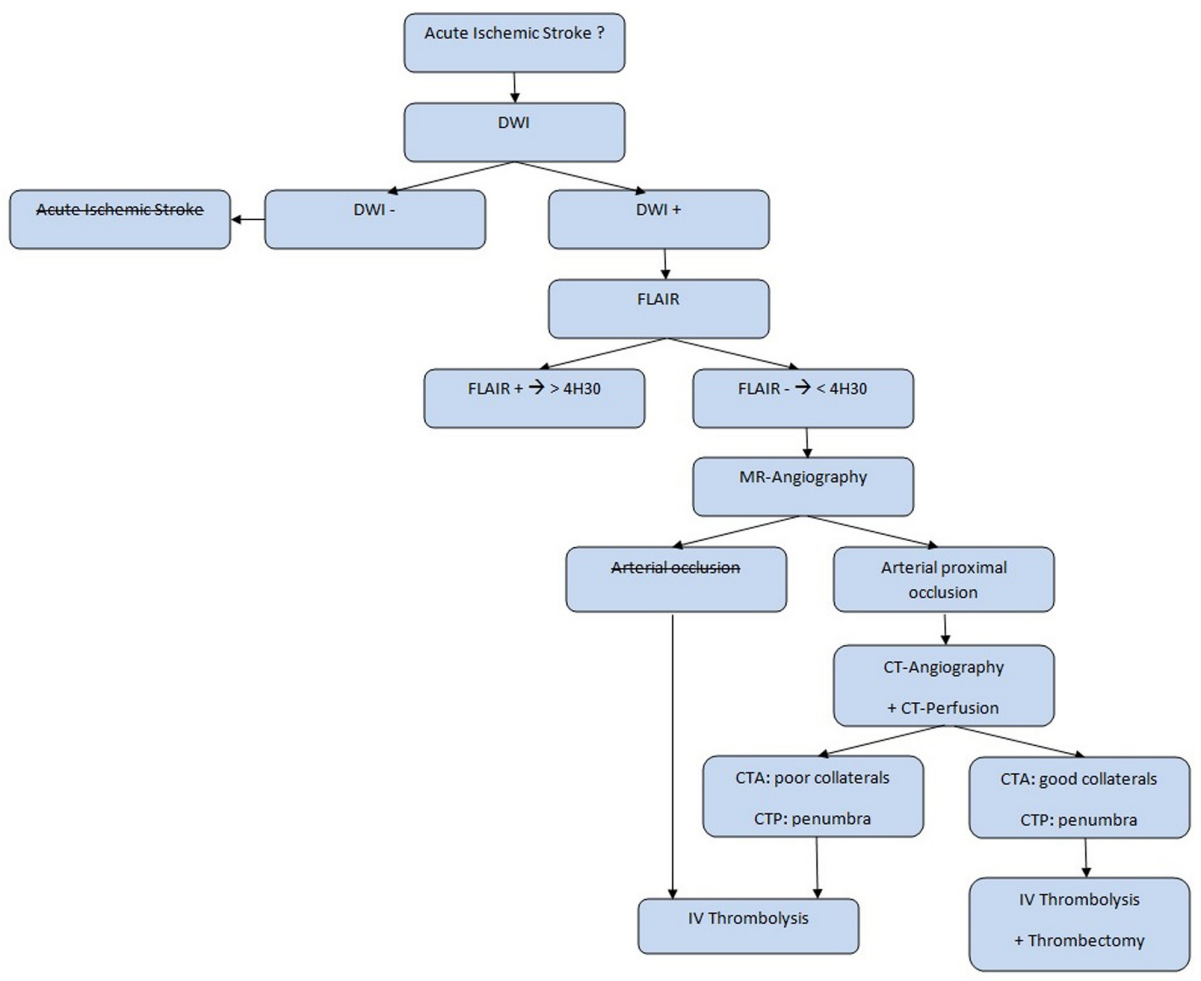
Acute wake-up stroke management algorithm.

Acute ischemic stroke imaging protocols vary depending on availability of MRI and post-processing software, local expertise, and institutional guidelines. Here below is the simplified algorithm for acute wake-up stroke management in our institution where we do not yet dispose of the ‘Rapid’ software (Figure 3).

